# Macroglial diversity: white and grey areas and relevance to remyelination

**DOI:** 10.1007/s00018-020-03586-9

**Published:** 2020-07-09

**Authors:** Inge L. Werkman, Dennis H. Lentferink, Wia Baron

**Affiliations:** 1 Department of Biomedical Sciences of Cells and Systems, Section Molecular Neurobiology, University of Groningen, University Medical Center Groningen, A. Deusinglaan 1, 9713 AV Groningen, the Netherlands; 2 Present Address: Department of Biology, University of Virginia, Charlottesville, VA 22904 USA

**Keywords:** Astrocyte, Plasticity, Heterogeneity, Oligodendrocyte, Remyelination

## Abstract

Macroglia, comprising astrocytes and oligodendroglial lineage cells, have long been regarded as uniform cell types of the central nervous system (CNS). Although regional morphological differences between these cell types were initially described after their identification a century ago, these differences were largely ignored. Recently, accumulating evidence suggests that macroglial cells form distinct populations throughout the CNS, based on both functional and morphological features. Moreover, with the use of refined techniques including single-cell and single-nucleus RNA sequencing, additional evidence is emerging for regional macroglial heterogeneity at the transcriptional level. In parallel, several studies revealed the existence of regional differences in remyelination capacity between CNS grey and white matter areas, both in experimental models for successful remyelination as well as in the chronic demyelinating disease multiple sclerosis (MS). In this review, we provide an overview of the diversity in oligodendroglial lineage cells and astrocytes from the grey and white matter, as well as their interplay in health and upon demyelination and successful remyelination. In addition, we discuss the implications of regional macroglial diversity for remyelination in light of its failure in MS. Since the etiology of MS remains unknown and only disease-modifying treatments altering the immune response are available for MS, the elucidation of macroglial diversity in grey and white matter and its putative contribution to the observed difference in remyelination efficiency between these regions may open therapeutic avenues aimed at enhancing endogenous remyelination in either area.

## Introduction

Multiple sclerosis (MS) is a chronic demyelinating disease of the central nervous system (CNS) characterized by inflammation [1], astrogliosis [2], and neurodegeneration [3–6]. MS can manifest in different disease courses, most commonly starting with relapsing–remitting MS (RRMS), which is characterized by inflammation-mediated exacerbations related to acute demyelination in the CNS and subsequent recovery. MS may also present in a progressive form in the absence of remission, either initially as in primary progressive MS (PPMS), or following RRMS, called secondary progressive MS (SPMS). Neurodegeneration, caused in part by ultimate failure of remyelination, is an underlying cause of disease progression [3–6]. Treatments for MS are limited to disease-modifying treatments that reduce inflammation, while a regenerative treatment overcoming remyelination failure is currently unavailable. MS heterogeneity is also reflected in differences in pathology between different CNS regions, which is best studied in leukocortical lesions that span both grey matter (GM) and white matter (WM). For example, in leukocortical lesions, remyelination is more robust in the GM part than in its WM counterpart and differences in cellular density and activation are observed [7, 8]. This diversity in cellular identity and/or responses may underlie regional differences in remyelination, and although remyelination may occur in these lesions, remyelination is often insufficient in either area [9].

The CNS predominantly consists of neurons, microglia and macroglia, the latter comprising astrocytes (ASTRs) and oligodendroglia, i.e., myelin-forming oligodendrocytes (OLGs) and OLG progenitor cells (OPCs). In the adult human brain, the ratio of glial cells to neurons is ~ 1:1 or even smaller [10, 11], unlike a ~ 10:1 ratio, as previously commonly reported in the literature [12] and textbooks ([13]; reviewed in [11]). The CNS can be grossly divided into two regions, GM and WM. GM contains mainly neuronal cell bodies, dendrites and axon terminals, whereas axons primarily reside in WM. Thus, synapses are more prominent in GM areas, while WM areas have a higher myelin content. Also, the abundance of OLGs and ASTRs in the CNS is not uniform and is region dependent. In most adult human brain regions, OLGs are the most numerous of glial cells, with a percentage ranging from 29% in the visual cortex [11, 14], to 75% in the neocortex [11, 15, 16]. When comparing their abundance in GM and WM of the human frontal cortex, OLGs are more numerous among the glial cells in the WM (69% versus 36.6% of glial cells) [11, 17]. ASTRs follow OLGs in numbers in most brain areas, such as in the frontal cortex WM (24% of glial cells), but not in the frontal cortex GM, where they outnumber OLGs (46.5% of glial cells) [11, 17]. What determines these homeostatic cell densities in distinct brain regions and what the functional relevance is of these differences are still open questions.

Over the past years, accumulating evidence indicates that macroglia from the GM and WM display regional plasticity and intrinsic heterogeneity, the first being adaptations of the same cell type to the local functional needs and responses to injury, and the latter being intrinsic transcriptional differences in cell populations [18]. These regional differences will have consequences for cell functioning upon CNS injury, such as demyelination and remyelination. Indeed, like observed in leukocortical MS lesions, in the cortex (GM area), remyelination is more efficient upon toxin-induced demyelination in experimental models for successful remyelination than in the corpus callosum (WM area) [19, 20]. Here, we review the current literature on the diversity of macroglial cells, and discuss how this may contribute to regional differences in successful remyelination and upon remyelination failure. We will start with an introduction to macroglia, followed by a detailed overview on the topic of macroglial diversity in the healthy CNS, focusing on GM and WM. Next, we discuss macroglial diversity in the context of regional differences in successful remyelination, and in light of remyelination failure and its implications for MS. Overall, this review recommends to take regional differences into account when developing and/or assessing remyelination-based treatments for MS.

## Introduction to macroglia

### Oligodendroglial lineage cells

OLGs ensheath axons with myelin, which is a tight stack of several phospholipid bilayers that provides metabolic support to axons [21] and facilitates rapid saltatory conduction of nerve impulses [22, 23]. In addition, oligodendroglial lineage cells are involved in synapse modulation and neurotransmission in both GM and WM [24, 25]. Oligodendroglial lineage markers include the transcription factors OLIG2 and SOX10. Mature OLGs develop from OPCs, which are PDGFRα and NG2 (also known as CSPG4)-expressing cells that comprise ~ 5% of the adult rodent CNS [26–28]. Of note, PDGFRα and NG2 are co-expressed on > 99.5% of non-vascular cells in the rodent CNS [29, 30]. Upon maturation, these cells pass an immature, pre-myelinating stage that can be identified by the transient expression of BCAS1 and ENPP6 [31, 32]. At this intermediate pre-myelinating stage, the myelin lipids sulfatide and galactosylceramide are already present at the cell surface. Myelinating OLGs are recognized by their expression of myelin-specific proteins of which MBP and PLP are the major ones [33–35].

The process of developmental oligodendrogenesis and subsequent myelination is well studied in rodents. In an elegant fate mapping study, Kessaris and colleagues [36] showed that OPCs are derived from neural progenitors called radial glia and populate the murine brain in three waves. At embryonic day 11.5 (E11.5), a first wave of OPCs emerges from the medial ganglionic eminence and anterior entopeduncular area. A second wave is generated from the lateral and/or caudal ganglionic eminences at E15. OPCs that emerge from both waves populate the murine cerebrum in a ventral to dorsal manner [36]. The third wave of OPCs occurs in the first week after birth and originates from the dorsal cortex. Remarkably, OPCs that are derived from the first wave disappear after birth and are virtually undetectable in adulthood [36]. Subsequent developmental myelination is a  highly orchestrated process. First, OPCs proliferate [37] and migrate towards naked axons [38]. There, OPCs differentiate into pre-myelinating OLGs and extend multiple processes that contact axons but do not yet myelinate. Upon withdrawal of mainly axon-derived inhibitory factors for OLG differentiation (reviewed in [39]), pre-myelinating OLGs retract their secondary and tertiary processes and myelin membranes are elaborated from the tips of the primary processes. These myelin membranes enwrap receptive axons multiple times, followed by the formation of compact myelin via cytoplasmic and exoplasmic reduction [40]. During myelin biogenesis, OLGs synthesize considerable amounts of myelin components, such as MBP, PLP, galactosylceramide and cholesterol, which can take up to 100 times the weight of the cell [41]. In fact, OLGs have the highest oxidative metabolism of all cells in the CNS during active myelination [12, 42]. Additionally, levels of the anti-oxidant glutathione are remarkably low in OLGs [43]. These features might explain why myelinating OLGs are exceptionally vulnerable to metabolic stress [44], possibly contributing to the multitude of pathologies involving demyelination.

Each OPC occupies an individual niche that is maintained by self-avoidance [45]. These OPCs can proliferate in the adult CNS of both rodents and humans [28, 45–48]. Notably, OPCs in the adult brain differ from developmental OPCs; adult OPCs are bound by the O4 antibody which recognizes sulfatide, have longer cell cycle times, slower migration rates, longer duration of maturation, and lower responsiveness to growth factors [49–53]. Surprisingly, murine adult OPCs differentially express 2361 genes compared to neonatal OPCs, while in adult OPCs only 37 genes are differentially expressed compared to OLGs [54]. This indicates that based on their transcription profiles, adult OPCs look more like myelinating OLGs than neonatal OPCs. In line with this, a recent study that compared human OLGs in development and aging revealed that based on gene expression, a distinction can be made between OPCs from pediatric and adult brains [55]. More specifically, gene ontology annotations enriched in OPCs in the pediatric human brain are related to OLG differentiation, extracellular matrix (ECM) metabolism, axon guidance and cholesterol transport, while gene ontology annotations enriched in OPCs in the adult human brain are related to regulation of cell projections, regulation of molecular transport, and superoxide metabolism [55]. In addition, rodent adult OPCs in the aged CNS have increased DNA damage and decreased metabolic function and fail to respond to differentiation signals both in vitro and in vivo [56]. This may underlie the poor remyelination observed in aged rodents [56].

### Astrocytes

ASTRs have a plethora of functions, including providing trophic support to neurons, regulating synapse formation and pruning, maintaining the integrity of the blood–brain barrier (BBB) [57–60].  ASTRs also play a direct role in the formation of myelin membranes by supplying lipids to OLGs [61, 62]. During development in rodents, most ASTRs are formed after the generation of neurons and OPCs from radial glia [63–66]. Radial glia are a heterogeneous population of cells which is formed based on a spatial and temporal patterning program in a columnar organization [63, 65, 66]. While OPCs are derived mostly from the motor neuron progenitor (pMN) domain [63–66], three populations of ASTRs originate and migrate from the progenitor domains p1, p2 and p3, with p1 being the most dorsal and p3 being the most ventral domain [64]. In rodents, the first ASTRs are detected at embryonic day 16 [65]. After asymmetrical migration of newly formed ASTRs, the number of ASTRs largely increase in the brain by  local symmetrical division  [66, 67]. The vast majority of ASTRs are formed during the first month after birth, when the ASTR population increases 6- to 8-fold [66, 68], but in contrast to OPCs, postnatal (re)distribution of ASTRs does not occur [65, 69, 70]. The final ASTR phenotype is thought to depend on its local cellular environment as well as on the region-specific functional demands [63, 65, 66]. Markers of immature ASTRs include Fabp7/Blbp and Fgfr3 [66, 71–74], and mature ASTR markers include Aldh1l1, S100B, Aldoc, Acsgb1, and Pla2 [66, 75]. However, there is no uniform ASTR surface marker that labels all ASTRs, which complicates the isolation of the complete ASTR population from unlabeled (human) tissue. Astrocytogenesis is promoted by Sox9 and Nifa/b [76], with Sox9 being especially important for ASTR development in GM [77]. This suggests that Sox9 may have a possible role in ASTR diversification [76, 77]. ASTRs are further characterized by the presence of filamentous proteins, including vimentin, desmin, synemin, and glial fibrillary acidic protein (GFAP) [78–81], of which GFAP is the most abundant [82–84]. In postnatal week 3, ASTRs are considered to be morphologically mature [85] and further aging of murine ASTRs does not induce major changes in their homeostatic and neurotransmission-regulating genes [75, 86]. However, ASTRs go into senescence [87], and aged murine ASTRs upregulate genes involved in synapse elimination and downregulate genes related to mitochondrial function and anti-oxidant capacity [86]. Moreover, upon aging, ASTRs acquire a more pro-inflammatory phenotype [86, 88]. The functional consequences of these age-related changes are not completely understood yet.

**Fig. 1:**
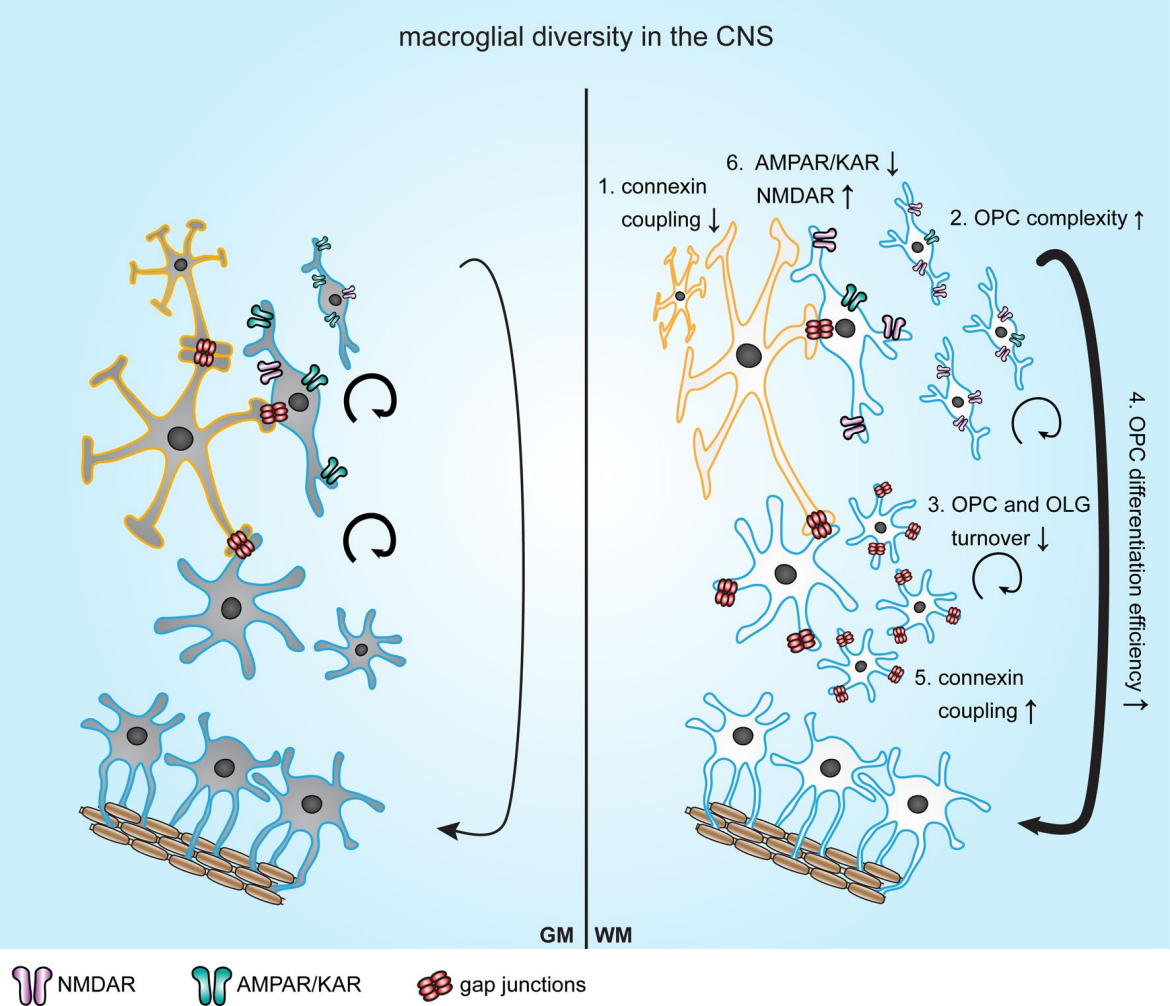
Schematic representation of macroglial diversity in grey and white matter areas of the central nervous system (CNS). Protoplasmic astrocytes (ASTRs) reside in the grey matter (GM) and are highly connected via gap junctional coupling to other protoplasmic ASTRs via connexin (Cx)43 and Cx30. Fibrous ASTRs are mainly present in the white matter (WM), have limited coupling to other fibrous ASTRs and only express Cx43 (**1**, [185–187]). Oligodendrocyte progenitor cells (OPCs) in GM are morphologically less complex than OPCs that reside in WM (**2**, [90, 103]). Furthermore, more OPCs ( [7, 30, 110]) and oligodendrocytes (OLGs; [11, 17]) are present in WM, but the turnover of both OPCs and OLGs (**3**, [133]) is lower in WM than in GM. OPCs differentiate more efficiently in WM than in GM (**4**, [94, 95, 99, 101]). OLGs express Cx32 and Cx47, which make heterotypic gap junctions with Cx30 and Cx43 on ASTRs, respectively, that are particular important for developmental myelination and OLG survival in WM (**5**, [183, 186, 189–191]. Finally, OPCs in GM display higher numbers in AMPA/kainate receptors (AMPARs/KAR)s, while NMDA receptors (NMDARs) are more abundant on wmOPCs (**6**, [52]). ASTRs are indicated with a yellow border and oligodendroglial lineage cells with a blue border

In conclusion, macroglia develop sequentially from radial glia during development, and obtain age-related changes in their phenotype and transcriptional profile. In addition, recent evidence demonstrates that macroglia from different regions appear as diverse populations throughout the CNS. In the following section, current knowledge on the regional diversity of OPCs, OLGs and ASTRs in GM and WM areas of healthy CNS will be outlined (summarized in Fig. 1).

## Diversity of oligodendroglial lineage cells

### Heterogeneity of oligodendrocyte progenitor cells in grey and white matter

Adult OPCs are scattered throughout the brain, but are more abundant in the corpus callosum (~ 120 cells/mm^2^ or 8% of cells) than in the cortex (~ 80 cells/mm^2^ or 3% of cells) of young adult mice [30]. In 2002, a study reported that OPC formation in the cortex was affected more by mutations in PLP or its splice variant DM20, than OPC formation in the corpus callosum. This indicates that during development oligodendrogenesis is differentially regulated between GM and WM [89]. A distinct regulation of developmental oligodendrogenesis in GM and WM is also observed upon conditional deletion of Smoothened, a regulator of sonic hedgehog (Shh) signaling, which results in temporal deletion of OPCs. Subsequently, OPCs in WM (wmOPCs) fully repopulate the depleted area, while recovery of OPCs in GM (gmOPCs) is limited [90]. This implies that gmOPCs are more dependent on Shh signaling for expansion. Subsequent studies in rodent models indicate that in vivo, wmOPCs mature more efficiently into myelinating OLGs than gmOPCs, which proliferate more slowly and produce fewer mature cells. However, survival of gmOPCs and wmOPCs is comparable [30, 48, 91–94]. Possibly as a consequence of this, OPC density in the adult rodent brain is higher in WM than in GM [16] (Fig. 1). Notably, the percentage of proliferating OPCs largely declines in WM after postnatal day 16, after which the OPC proportion that proliferates remains relatively stable. An ongoing more subtle decline in the proliferating OPC portion is observed in GM [52]. Ultimately, upon aging, the percentage of proliferative OPCs becomes similar in both GM and WM [52].

A transplantation study by Viganò and colleagues [95] also hinted at regional differences between OPCs derived from GM and WM. This study demonstrated that wmOPCs differentiate into OLGs equally well in both healthy GM and WM, whereas gmOPCs remain more immature irrespective of the environment. Hence, OPCs seem to carry a memory or intrinsic potential that is not altered by a new and different, healthy environment. In other words, gmOPCs and wmOPCs have functionally different phenotypes [95]. Indeed, OPCs display diversity in electrical properties [52, 96, 97], gene expression profiles [98–101], proliferation [51, 52, 101] and differentiation [94, 99, 101] rates, injury response [101–103], and other parameters [30, 104–106]. In vitro, rat postnatal day 2-derived gmOPCs are morphologically less complex, have less transcripts of common OLG-maturation genes, proliferate more in response to PDGF and FGF2, and differentiate slower than wmOPCs [101] (Fig. 1). In contrast, murine postnatal day 8-derived wmOPCs proliferate more in response to PDGF than gmOPCs [107], indicating that regulation of regional OPC proliferation depends on multiple factors including developmental timing and the presence of mitogen(s). Nonetheless, these findings indicate that wmOPCs are more mature than gmOPCs even after prolonged culture in vitro [94, 101]. That oligodendroglial lineage cells in the WM have a more complex phenotype in vitro is supported by an in vivo study describing that premyelinating OLGs in the corpus callosum have more processes and myelinate more axons in the developing rat brain at postnatal day 7 than premyelinating OLGs in the cortex [108]. Furthermore, in the rat cortex at postnatal day 50, NG2-positive OPCs present in a classical stellate form with processes radiating in all directions, while OPCs in the corpus callosum have an elongated morphology with multiple processes that follow axons. Additionally, OPCs in the rat corpus callosum produce longer processes than OPCs in the cortex [109]. In line with this, in the adult human brain, gmOPCs have a more regular network-like appearance than wmOPCs [110]. Other studies report differences in voltage-gated ion channels and spiking behavior of gmOPCs and wmOPCs [96]. More specifically, the density of AMPA/kainate receptors is higher on OPCs from the cortex, while on OPCs from the corpus callosum the density of NMDA receptors is higher at postnatal day 9 (Fig. 1). This observation may underlie the observed regional differences in proliferation and differentiation rates. As electrical activity is known to stimulate OPC proliferation either by stimulating the release of PDGF from neurons or making OPCs more responsive to PDGF [111], the shorter cell cycle time of wmOPCs may be explained by a higher density of voltage-gated potassium channels and subsequent higher peak outward current in WM [112, 113]. In turn, as NMDA receptors are involved in activity-dependent myelination [114, 115], the higher density of NMDA receptors on wmOPCs may contribute to their greater differentiation potential [52].

As OPC proliferation and differentiation are influenced by extrinsic factors, environmental cues may contribute to differences in OPC diversity. For example, more environmental signals that inhibit OPC proliferation and arrest their differentiation are present in GM than in WM, although it is unknown where these signals originate [30, 94, 116]. When developing rats are exposed to cuprizone, a copper chelator that causes specific depletion of OLGs, via a maternal diet from gestational day 6 to postnatal day 21, the density of oligodendroglial lineage cells is widely impaired in cortical regions at postnatal day 21, whereas only mature OLGs are affected in the corpus callosum [117]. An increased expression of the anti-aging protein Klotho may protect wmOPCs from cuprizone toxicity [118]. Conversely, while prenatal PDGFRα-positive OPCs display remarkable regional heterogeneity at the transcriptional level in mice, the transcriptional differences converge to a common region-independent profile upon transition to neonatal OPCs [119]. Single-cell RNA sequencing (scRNAseq) of murine CNS tissue from various brain regions from the developing and young adult murine brain revealed also a single OPC population independent of region or age [53]. However, OPCs in the developing murine brain display more transcriptional signs of proliferation than OPCs in the more mature murine brain [53]. In the same study, a differentiation-committed OPC (COP) population was identified that is slightly more abundant in the corpus callosum than in the somatosensory cortex [53], and may reflect a difference in maturation state of the region in the developing brain. Similarly, independent single-nucleus RNA sequencing (snRNAseq) studies on post-mortem human brain tissue identified only one OPC population in the adult brain [120, 121]. A recent scRNAseq study on ex vivo isolated oligodendroglial lineage cells from surgical material revealed two transcriptionally different OPC populations; an early OPC population present in fetal tissue and a late OPC population that is present in pediatric, adolescent and adult tissue [55]. Similar to what is known during murine brain development, genes related to cell cycle regulation were upregulated in the early OPC population [55]. Hence, although it has been suggested that OPCs arising from the different waves might be functionally different and myelinate specific brain regions [122], in the developing murine CNS, PDGFRα-positive OPCs generated before birth converge on a transcriptional level, i.e., postnatal OPCs from brain and spinal cord have an almost similar transcriptional profile [119]. However, at postnatal day 7, OPCs from the murine spinal cord are more mature than OPCs in the brain based on the expression of late-stage differentiation markers *Mog*, *Mag,* and *Mal* [119]. Also, in support of a single OPC population, are studies that demonstrate that OPCs derived from the three different waves initially present comparable electrophysiological capacities [52], but become regionally diverse postnatally. A similar acquired permanent regional segregation of OPCs is observed in the spinal cord of zebrafish. In zebrafish, OPCs are more quiescent when OPC cell bodies are present in neuron-rich areas, whereas OPC differentiation is favored when OPC cell bodies reside in axo-dendritic areas [123]. Hence, in zebrafish, the microenvironment where the OPC cell body resides determines its behavioral subtype and differentiation capacity [123]. This may resemble the observed differences in OPC differentiation capacity in GM and WM. Altogether, postnatal OPCs from different regions are first transcriptionally similar, and given their limited motility, segregate and acquire differences in protein expression and function via their local microenvironment.

### Heterogeneity of oligodendrocytes in grey and white matter

In the rodent CNS, OPC differentiation into myelinating OLGs continues up to 8 months after birth [30, 48, 94]. This differentiation can be initiated by, and is required for, the learning of complex tasks [124]. In humans, OLGs may be produced continuously although OPC proliferation declines with age [125, 126]. Like in rodents, the learning of a complex motor task induces myelin remodeling in humans [127, 128]. In mice, OLGs that reside in the GM show less morphological plasticity. More specifically, two very recent in vivo imaging studies [129, 130] revealed that cortical OLGs hardly remodel their compacted myelin segments, whereas compacted myelin segments in WM are thickened upon increased axonal activity [131] or can be elongated when a neighboring myelin segment is ablated in zebrafish [132]. In the human WM, OLG turnover is especially low and most OLGs are formed in the first decade of life with an annual turnover of ~ 1 in 300 OLGs (0.3%) [133]. This in contrast to adult human GM, where the expansion phase of OLGs appears to be much longer, up to the fourth decade of life; combined with an annual turnover of 2.5% [133].

**Fig. 2:**
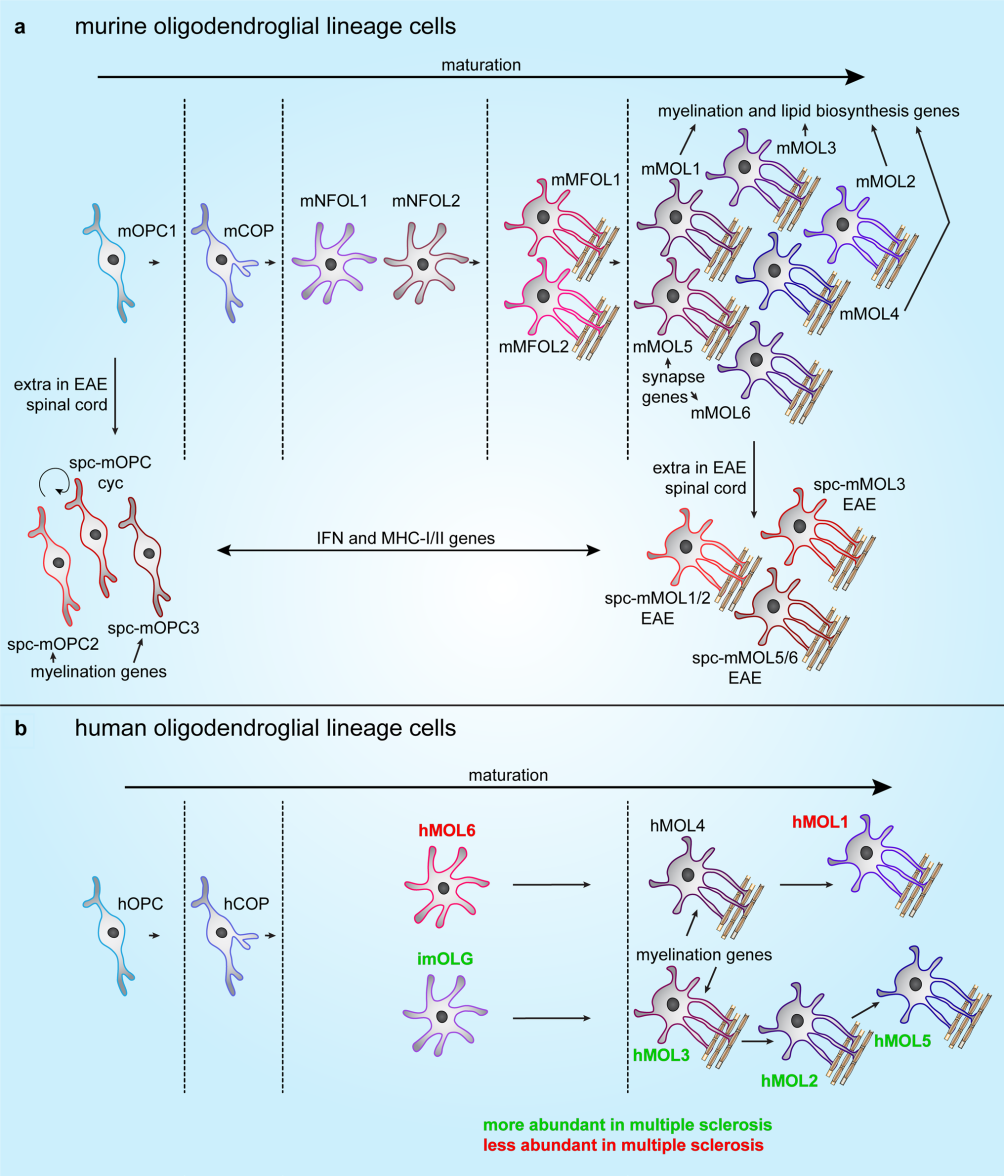
Schematic representation of transcriptionally distinct oligodendroglial lineage cell subpopulations in murine and human physiological and pathological conditions. **a** Single-cell RNA sequencing identified 12 oligodendroglial lineage cell subpopulations in ten different regions from the developing and adult murine central nervous system [53]. A single population of oligodendrocyte progenitor cells (mOPC1) differentiates into a single population of differentiation committed OPCs (mCOP). This is followed by two populations of newly-formed oligodendrocytes (mNFOL1/mNFOL2) and two populations of myelin membrane-forming oligodendrocytes (mMFOL1/mMFOL2). Real diversification, as opposed to sequential maturation stages, occurs in the last stage and is apparent as six mature oligodendrocyte populations (mMOL1-6). Of these, mMOL1-4 express myelination and lipid biosynthesis genes, while mMOL5 and mMOL6 express synapse related genes. Upon induction of experimental autoimmune encephalomyelitis (EAE), an animal model for inflammatory aspects of MS that mainly manifest in the spinal cord (spc), three additional OPC populations are observed, including cycling OPCs (spc-mOPC cyc) and spc-mOPC2/3. Furthermore, three additional mMOL populations are observed; spc-mMOL1/2 EAE, spc-mMOL3 EAE and spc-mMOL5/6 EAE [183]. Notably, all EAE-specific populations express IFN-, MHCI- and MHCII-related genes. **b** Single-nucleus RNA sequencing on human post-mortem tissue of healthy subjects and MS patients identified one OPC population (hOPC) followed by one COP population (hCOP) and one immature oligodendrocyte population (imhOLG), and an intermediate pre-myelinating, mature OLG population (hMOL6). Also in human, real diversification starts in the last maturation stage, with another five mature hMOL populations (hMOL1-5). Of the identified populations in human, imhOLG and hMOL2,3 and 5 are more abundant in multiple sclerosis (MS) tissue than in control tissue, while hMOL1 and hMOL6 are less abundant in MS tissue [121]. Of note, although mMOL and hMOL share similarities, this is not reflected by the same group number [53]. IFN, interferon; MHC-I/II, major histocompatibility complex class-I/II

Whether diversity of OLG phenotype can be branded as heterogeneity of oligodendroglial lineage cells or their plasticity, was recently reviewed by Foerster et al. [18]. Diversity of mature OLGs was first observed in the 1920s by Pio del Río-Hortega. Based on morphology, he described OLGs with small cell bodies and many fine processes that reside in both GM and WM, and three additional distinct subtypes that are restricted to WM [134, 135]. After this initial observation of the four morphological distinct mature OLG subpopulations, OLG heterogeneity was mostly ignored. Only recently more attention has been given to the diversity of OLGs [136]. The rise of sequencing technologies allows the study of transcriptomics and has provided a considerable contribution to the knowledge of regional heterogeneity of developing OLGs [137]. First, Zhang et al. [32] produced a detailed comparison of the transcriptome of the different cell types of the mouse cortex, including three oligodendroglial maturation stages. Zeisel et al. [138] performed quantitative single-cell analysis of the transcriptome on cells of the mouse primary somatosensory cortex and the hippocampal CA1 region [138]. This study demonstrates the possible existence of six OLG subpopulations based on gene expression that likely represent different maturation stages, of which one appears specific to the somatosensory cortex [138, 139]. scRNAseq on oligodendroglial cell types from various brain regions of the developing and young adult murine CNS categorizes 12 oligodendroglial lineage populations that include five different maturation stages, including one murine OPC stage (mOPC), one murine differentiation-committed (mCOP) stage, two murine newly-formed OLG stages (mNFOL), two murine myelin-forming OLG stages (mMFOL), and six murine mature OLG (mMOL) stages (Fig. 2a). Remarkably, of the six mMOL stages, mMOL1-4 are enriched in myelination genes and genes involved in lipid biosynthesis, while transcripts of synapse genes are enriched in mMOL5 and mMOL6 (Fig. 2a), both of which are predominantly present in the adult murine brain. In contrast to mOPCs, which are transcriptionally similar between brain regions, of the six mMOL populations the mMOL5 population is relatively enriched in the adult somatosensory cortex, and the corpus callosum has a relative enrichment in mMOL1, 4, 5 and 6 populations [53]. The identification of six different mMOL stages confirms heterogeneity of mature OLGs at the transcriptional level and their transcriptional profile indicates regional heterogeneity in mMOL function, including genes related to synaptic function instead of myelination in the cortex. Regional heterogeneity of mature OLGs may be acquired by the microenvironment upon differentiation-inducing cues [119], which is also previously described in human CNS development [140, 141].

Similarly, using snRNAseq, six populations of mature OLGs in the adult human brain WM can be distinguished, Oligo1 to Oligo6 [121]. Mature human OLG populations are from here on referred to as hMOL1 to hMOL6, as some shared similarities with the six defined mMOL populations are evident, not necessarily reflected by the same group number [53]. Two major developmental end-stages of hMOLs are identified by pseudo-time analysis; hMOL6 develop via hMOL4 into an end-stage hMOL1, and hMOL3 develop via hMOL2 into end-stage hMOL5 [121] (Fig. 2b). Surprisingly, myelination-related genes are highly expressed in the two intermediate populations hMOL3 and hMOL4, and not in the maturation endpoint populations [121] (Fig. 2b). This indicates that in addition to myelination, fully matured wmOLGs likely have other important functions not yet identified [53, 121] that may relate to myelin maintenance and/or function in synaptogenesis. Another possibility is that these two fully mature OLG populations may actively support neuronal function. OLGs provide trophic support to neurons [23], and OLGs that have formed myelin membranes actively transport glycolysis products from the blood stream to the myelinated axon via monocarboxylate transporters (MCT) 1 and 2 [21]. In addition, MCT1 in OLGs is required for neuronal survival and function [142]. Notably, in healthy brain tissue, hMOL6 are most abundant at the border between GM and WM [121]. While Jäkel et al. [121] solely studied WM brain tissue, in another recent snRNAseq study [120] GM, WM, and leukocortical MS lesions were analyzed and compared to tissue of healthy subjects. In this study, only one OPC population and OLG population were identified in healthy brain tissue. As this study focused on differences between healthy and MS brain tissue, the authors did not elaborate on potential differences between control GM and WM [120]. Hence, whether in humans a relative enrichment for one of the hMOLs in GM compared to WM or vice versa exists, remains to be determined.

Thus, in contrast to OPCs, mature OLGs not only differ in their morphology, but are also heterogeneous at the transcriptional level. As a consequence, the two divergent maturation hMOL patterns may have a different myelinogenic potential, i.e., differences in composition, or number and length of myelin segments. Although the myelinogenic potential of the mMOL and hMOL populations has not yet been addressed, the myelinogenic potential of OLGs in different brain regions in vivo has been described, which will be discussed next.

### Diversity in myelinogenic potential?

In vivo analysis of single cells revealed that OLGs in a given region display a great diversity in the number of myelin segments they elaborate, while the length of each myelin segment formed by an individual OLG also varies [143]. Although OLGs in the cerebral cortex form a slightly higher mean number of myelin segments per OLG and a seemingly shorter myelin segment length compared to OLGs in the corpus callosum, the myelinogenic potential appears not to be region-specific [143]. This indicates that the number and length of myelin segments is likely regulated by microenvironmental cues. In support of this, neuronal activity-mediated regulations of intracellular Ca^2+^ concentrations affect myelin sheath development [144]. Other factors that may affect the number of axons myelinated and the length of the myelin segments are axonal caliber and OPC competition. For example, compared to OLGs in the cerebellar WM, OLGs in the corpus callosum of the rat myelinate more axons (9.6 versus 6.7 axons on average) and have shorter myelin segments, (79.1 µm versus 106.1 µm) [145], likely because axons in the corpus callosum have a smaller diameter than those in the cerebellar WM [146]. In line with this observation, studies in rodents and cats demonstrate that larger axons provoke the production of longer, but fewer, myelin segments by OLGs [147–150]. Moreover, the density of OPCs also regulates the myelinogenic potential. The abundance of OPCs has a negative correlation with the number of myelin segments, a process mediated via Nogo-A [143]. In addition, OLGs that myelinate nanofibers in vitro adapt myelination patterns to the nanofiber diameter, i.e., the myelin sheath length increases with nanofiber diameter [151]. It is hypothesized that adapting myelination to axonal size is an evolved trait [145]. Motor output, which is critical for fast reactions upon threats, requires higher conduction speed than less critical data movement between the cerebral cortices. Hence, the first is signaled over thicker, and the latter over thinner, axons [145]. This evolutionary advantage might also underlie: (1) the differences in myelination-level of the adult CNS, i.e., the optic nerve consists of almost only myelinated axons [152] and the cortex and corpus callosum contain both myelinated and unmyelinated axons [153], and (2) the timing and duration of myelination as suggested by neuroimaging and cell age studies [154, 155]. For example, in humans, the volume of WM increases up to 19 years of age, while myelination of GM areas is not complete until the fourth decade of life [156]. The number of OLGs in mice is almost twofold higher in the corpus callosum than in the almost completely myelinated optic nerve, while OLG survival in these regions is comparable [48]. This is possibly due to a higher amount of myelination-stimulating signals from the higher number of naked receptive axons [48].

Not only the number of naked axons differs between GM and WM, also the direction of these axons. Thus, while in the axon bundles of WM tracts myelination is characterized by OLG processes that align with axons, the orientation of myelin segments in the GM is more omnidirectional as axons in the GM are not uniformly aligned [157]. On the other hand, the source of OLGs influences their myelination pattern, i.e., cultured OLGs derived from the spinal cord generate larger myelin sheaths than OLGs from the cortex [151], pointing also to intrinsic differences in OLG maturation from different regions. Differences between myelination during development and in the adult CNS have been observed as well. More specifically, myelinating OLGs that have developed in the optic nerve during adulthood have more and shorter myelin segments than OLGs formed during early development [48]. Possibly, newly-produced OLGs in the adult brain either replace dying OLGs or incorporate between the pre-existing myelin segments and in this way, the total number of contributing OLGs increases [48]. While it is likely that axonal signals that determine myelin segment length and thickness are lacking or less prominently present in the adult than in the developing CNS, it cannot be excluded that reported differences between neonatal and adult OPCs may contribute [54, 106].

Whether myelin composition differs between different regions has not been thoroughly analyzed yet. It has been observed that human WM homogenates, i.e., that contain cells and myelin, are relatively enriched in lipid content (54.9% in WM versus 32.7% in GM), while human GM homogenates are more enriched in protein (55.3% in GM versus 39.0% in WM). Notably, fatty acids such as ethanolamine and serine glycerophosphatides, and lecithin are more abundant in GM than in WM homogenates, while cholesterol, sulfatide and cerebroside levels are higher in the WM lipid fraction [158, 159]. Whether this reflects the lower myelin content in GM or differences in myelin composition, and thus heterogeneity of GM and WM myelin per se, remains to be determined. In favor of the latter, myelin protein concentration and myelin protein activity from distinct human brain regions differ more than the regional difference in myelin content accounts for [160]. Although the concentration of PLP, MBP and activity of CNP is higher in WM homogenates than in their GM counterparts, the fold difference ranges between 3.3 × for CNP (frontal GM versus WM) and 9.6 × for MBP (frontal GM versus WM), which may point to a regional heterogeneity in myelin composition (Table 1). This hypothesis is supported by differences in the lipid percentage ratio which differs between 1.3 × (GM *versus* WM) for cholesterol and 3.7 × (GM *versus* WM) for cerebroside (Table 1). Plasticity of myelin is also observed during aging. The abundance of MBP decreases in healthy human aging [161, 162] and even more in patients with Alzheimer’s disease [163]. In contrast, in aged rhesus monkeys, MBP levels remain unchanged, whereas CNP levels increase [164]. How this plasticity in composition affects the quality of myelin is not yet known and whether this is different among species are interesting areas of future research.

**Table 1:** Regional concentrations or activity of myelin proteins and lipids (adapted from [158, 159])

	Frontal GM	Frontal WM	FD^a^	Temporal GM	Temporal WM	FD^a^	Corpus callosum
*Protein*							
PLP (µg/mg protein)	95.8	488.8	**5.1**	48.2	398.2	**8.3**	516.8
MBP (µg/mg protein)	22.8	218.0	**9.6**	23.5	155.5	**6.6**	178.2
CNP (U/mg protein)	4.7	15.4	**3.3**	3.5	16.2	**4.6**	15.5

	GM^b^	WM^b^	FD^a^				
*Lipid*							
Cholesterol (% of total dry weight)	22.0	27.5	**1.3**				
Cerebroside (% of total dry weight)	5.4	19.8	**3.7**				
Sulfatide (% of total dry weight)	1.7	5.4	**3.2**				

^a^Fold difference (FD) in concentration, activity, or percentage between regional grey matter (GM) and white matter (WM)

^b^Specific region of GM and WM is unspecified [158]

Taken together, while OPCs are transcriptionally less diverse, mature OLGs intrinsically differ and constitute a heterogeneous group of locally established cells (Fig. 2). The diversity in OLGs may determine differences between myelination efficiency in GM and WM. Indeed, whereas oligodendroglial lineage cells continuously produce myelinating OLGs in WM, in GM, the majority of oligodendroglial lineage cells remain in an immature NG2-positive stage [94]. Whether the variety in myelin phenotype may also be a product of intrinsic differences in the myelin-producing cell, i.e., the OLG, in conjunction with axonal cues that orchestrate differences in myelinogenic potential, remains to be investigated. In addition to axonal cues, local cues of other cell types, such as regionally diverse ASTRs, may also affect the diversity of oligodendroglial lineage cells during development, aging or upon response to demyelinating injury.

## Astrocyte diversity

### Astrocyte subtypes in grey and white matter of the adult brain

Originally, ASTRs are divided into two groups based on their morphology and this relates to region; protoplasmic ASTRs are present in GM (gmASTRs) and fibrous ASTRs mainly reside in WM (wmASTRs) [165–167] (Fig. 1). Protoplasmic ASTRs are morphologically complex with a high number of fine processes that ensheath synapses and usually have one or two processes in contact with the microvasculature. Fibrous ASTRs are less complex and have long and thin processes with only a few branches, yielding a star-like appearance [78]. This morphological difference is accompanied by a more abundant presence of the intermediate filament protein GFAP in wmASTRs compared to gmASTRs [168]. The distinct ASTR subtypes may relate to their distinct function in either area. For example, fibrous ASTRs and protoplasmic ASTRs differ in their handling of glutamate [169, 170]. Also, protoplasmic ASTRs are evenly distributed throughout the cortex and bear their own microdomain with hardly any overlap between neighboring cells [171, 172]. Even though the exact role of microdomain organization is not clear, its architecture suggests a prominent role in coordination of synaptic activity and blood flow, potentially independent of neuronal metabolic activity [173]. In fact, each rodent protoplasmic ASTR covers between ~ 20,000 and 120,000 synapses, whereas a human gmASTR can cover ~ 270,000–2 million synapses [171, 174], which may improve memory and learning [175]. In addition, in the rodent brain, capillary density and branching is 3–5 times higher in GM than in WM [176, 177], which is accompanied by a lower BBB permeability in GM than in WM [178]. In contrast, fibrous ASTRs seem specialized in providing structural support for myelinated axons, as they have numerous overlapping processes combined with evenly spaced cell bodies [174]. Fibrous ASTRs are organized along WM tracts and longitudinally oriented in the plane of fiber bundles. Moreover, fibrous ASTRs also make contact with blood vessels and with nodes of Ranvier, where they modulate myelin thickness and conduction velocity [174, 179].

The classification of ASTRs into protoplasmic and fibrous ASTRs may be a simplified representation of ASTR subtypes. After the early discovery of ASTRs in 1913, Cajal divided ASTRs into different subclasses with a staining method using gold chloride that visualized both ASTRs and neurons, and classified ASTRs based on their morphology and contact with blood vessels [165, 180]. In 2006, an in depth morphological and biochemical analysis by Emsley and Macklis [181] divided ASTRs into nine different classes based on morphology, GFAP, and S100B expression. Adding to the complexity of ASTR form and functions, human and primate ASTRs are 2.6-fold larger in diameter and 15.6-fold larger in volume compared to rodent ASTRs [182]. As this increase in size is valid for both fibrous and protoplasmic ASTRs, this may represent an evolutionary optimal increase relative to the increase in total brain size [182]. Also, human ASTRs extend tenfold more GFAP-positive primary processes than their rodent counterparts [182]. Primates and humans have more subtypes of ASTRs than other mammals. Primates harbor two extra types of glia in the cortex; interlaminar ASTRs and varicose projection ASTRs [174]. It is hypothesized that these two ASTR subtypes provide a network for the long-distance coordination of intracortical communication thresholds and play a role in coordinating blood flow [174].

Although many different morphological and functional subtypes of ASTRs are described, in murine scRNAseq and human snRNAseq studies on WM, only two to three groups of transcriptionally different ASTRs are defined [121, 138, 183]. This is based on specific marker expression like *Gfap* and *Mfge8* in mice [138]*, GPC5* for human gmASTRs, and *CD44* for human wmASTRs [120]. Using reporter mice and a fluorescence-activated cell sorting panel of 81 cell surface antigens, Lin et al. [184] described five different ASTR populations based on ASTRs isolated from cortex, cerebellum, brainstem, olfactory bulb, thalamus, and spinal cord. These five populations displayed, in addition to a distinct surface antigen expression, also functional differences. Gene expression profiling revealed that although the five ASTR populations were functionally and morphologically different, three of the five populations were transcriptionally similar, indicating ASTR plasticity of a transcriptionally comparable population. Therefore, combined with the other two transcriptionally distinct populations, and consistent with RNAseq studies, three intrinsic, transcriptionally heterogeneous populations were described in this study. Of these, one population was more abundant in the cortex [184]. Hence, diversity of form and function is not solely based on intrinsic transcriptional heterogeneity, but may also derive from ASTR plasticity. Finally, ASTR density also varies between different brain regions. In mice, the density of ASTRs is highest in the subventricular zone (2500 cells/mm^2^) and ASTRs in the corpus callosum are more dense than ASTRs in the cortex (~ 80 *versus* ~ 10 cells/mm^2^) [181], indicating that different local functional demands require different numbers of ASTRs.

### Astrocyte coupling in grey and white matter

ASTRs are connected to each other by homotypic gap junction coupling via connexin 43 (Cx43), which is expressed in both gmASTRs and wmASTRs, and to a lower extent via Cx30, which is only expressed in gmASTRs [185, 186] (Fig. 1). In rodents, dye injection experiments indicate that the coupling between ASTRs in GM and WM significantly differs. In the cortex, on average, 94 ASTRs are coupled with a span of 390 μm in diameter [187], while in the corpus callosum ASTRs are coupled to few or no other ASTRs [187]. In contrast, a high degree of coupling between ASTRs is found in the optic nerve, with a coupling of 91% of the cells [188], indicating a large variety in coupling ability of wmASTRs [169]. Mature OLGs express Cx32 and Cx47, which make heterotypic gap junctions with Cx30 and Cx43 on ASTRs, respectively. Although both gmOLGs and wmOLGs express Cx32 and Cx47, their expression is higher in wmOLGs [189]. The coupling of ASTRs/ASTRs as well as the coupling ASTRs/OLGs increases during development [190]. ASTR Cx43 coupling to OLGs may be involved in myelin maintenance and is suggested to play a role in redistribution of potassium after neuronal activity. Indeed, OLG gap junction ablation [191–193] and/or the deletion of potassium channel Kir4.1 in OLGs [192, 194, 195] causes vacuolation of myelin. Gap junctions between ASTRs and OLGs are also crucial for developmental myelination and survival of OLGs [193, 196, 197], particularly in WM [198]. In mice, a double knock-out of astrocytic Cx43 and Cx30 results in widespread pathology of WM tracts during development that persists with aging, and includes vacuolated OLGs and intramyelinic edema [198]. In contrast, GM pathology was only observed in part of the hippocampus and restricted to edematous ASTRs. Thus, gap junctions between ASTRs and OLGs seem less important for OLG survival and myelin maintenance in GM [198], which may be reflected in the lower expression of Cx32 and Cx47 in gmOLGs [189].

Taken together, based on gene expression, morphology, and function, a variety of ASTR phenotypes can be discerned with region as an important determinant. ASTRs are one of the first responders to CNS injury, and upon demyelination, ASTR subtypes may differ functionally and differentially respond in GM versus WM. In turn, OPCs and mature OLGs from different regions may act differently in response to alterations in their microenvironment, including to response-induced alterations in ASTR-derived signaling factors, and their ability to remyelinate, which will be reviewed next.

## Macroglial diversity upon central nervous system demyelination and remyelination in rodent models

### Remyelination in grey and white matter

Regional differences in macroglia affect cells’ responses towards injury, and may therefore play an important role in the extent of disease pathology and recovery. For example, ASTR-mediated trafficking of mercury via gap junctions may result in uptake of mercury in gmOPCs, but not wmOPCs [199]. A valuable model to study regional diversity in macroglial responses upon demyelinating CNS is the dietary cuprizone model [200]. In adult mice, cuprizone feeding leads to reversible global demyelination in GM and WM of which the cortex and corpus callosum are most studied [200]. As spontaneous and robust remyelination is observed following withdrawal of the toxin, this model has provided insight in the process of remyelination. Upon demyelination in rodents, OPCs are transcriptionally activated and recruited to the area of demyelination, where they differentiate into myelinating OLGs, a process orchestrated by signaling from local microglia and ASTRs [201]. When administered to adolescent mice, cuprizone induces a different de- and remyelination phenotype in GM and WM. More specifically, the initiation and peak of complete demyelination is delayed in the cortex compared to the corpus callosum [19]. Several studies report that remyelination is more efficient in the corpus callosum than in the cortex upon cuprizone intoxication [202, 203]. However, limitations of the cuprizone model are that after initial demyelination, myelin debris clearance parallels the early processes of remyelination, i.e., mature OLGs appear regardless of whether the cuprizone diet is maintained or not [204]. Therefore, as demyelination is delayed in the cortex [19], likely also the re-expression of myelin proteins as well as remyelination are delayed in the cortex [19], preventing the comparison of regional differences in remyelination upon cuprizone feeding alone. However, upon co-administration of cuprizone and rapamycin, the remyelination process does not occur until treatment cessation [20]. Under these conditions, when remyelination starts at the same time in GM and WM, remyelination proceeds faster in the cortex than in the corpus callosum [20]. Hence, the timing of demyelination and efficiency of remyelination are distinct between GM and WM. Notably, the differences in the time-course of de- and remyelination is also a heterogeneous process within GM itself; upon cuprizone-induced demyelination, the timing and speed of remyelination differs between the cingulate cortex and the GM of the hippocampus [203, 205]. Whether regional diversity of local macroglial responses may contribute to more efficient remyelination in GM than in WM is discussed next.

### Oligodendrocyte progenitor cell diversity and remyelination

**Fig. 3:**
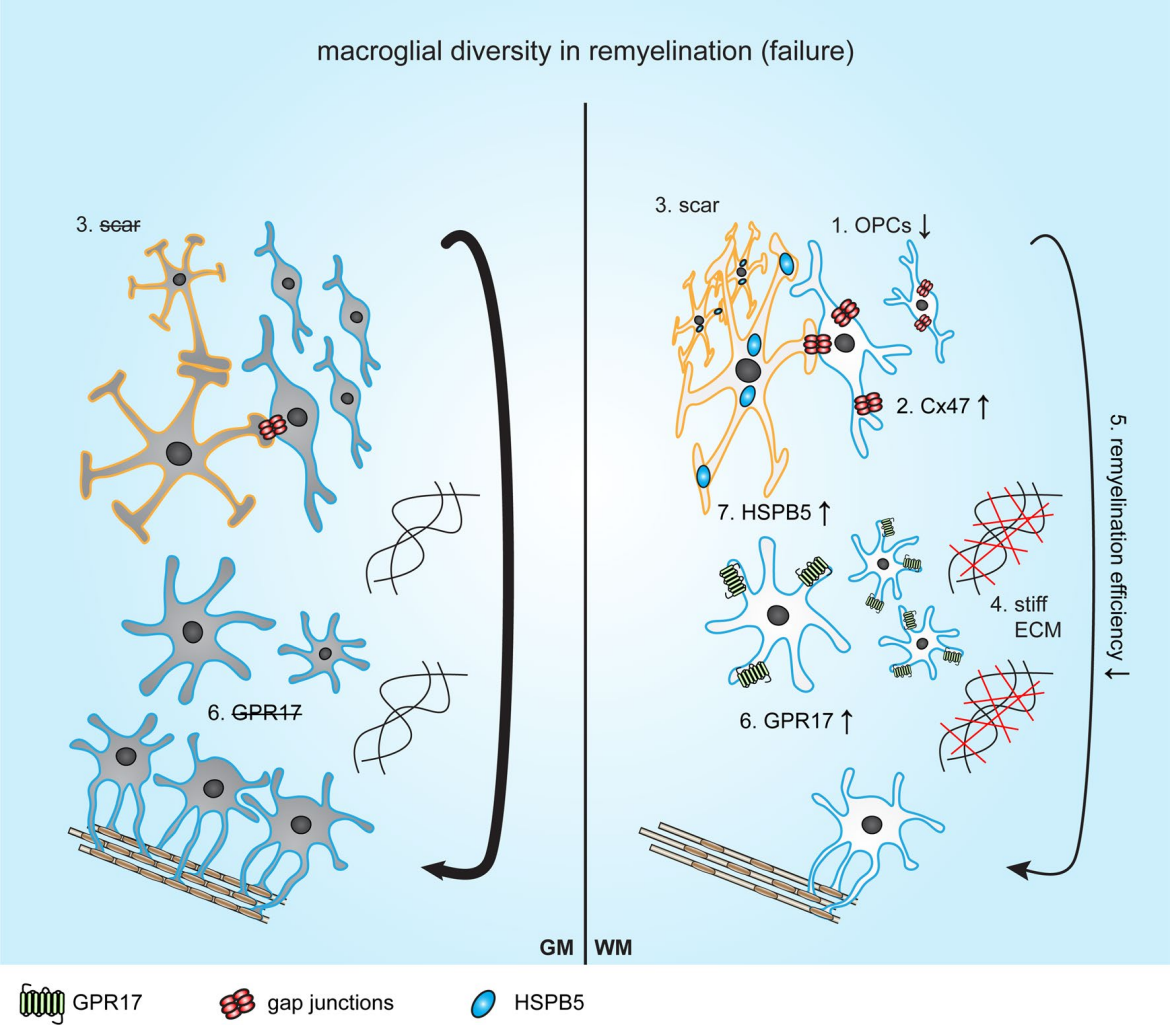
Schematic representation of macroglial diversity and its role in remyelination (failure) in grey matter and white matter (multiple sclerosis) lesions. Oligodendrocyte progenitor cells (OPCs) are more abundant in grey matter (GM) multiple sclerosis (MS) lesions than in white matter (WM) MS lesions (**1** [7, 8]). An increase of connexin (Cx) 47 on OPCs is observed in normal appearing white matter (NAWM) (**2** [312, 313]). Astroglial (ASTR) scar formation is observed in WM but not in GM MS lesions (**3** [7, 289, 290]) and the ECM becomes more stiff in WM MS lesions (**4** [235]). Overall, both upon toxin-induced demyelination and in MS, GM lesions remyelinate more robustly than WM lesions (**5** [7, 8]). Upon toxin-induced demyelination the expression of G-protein coupled receptor 17 (GPR17) is induced on wmOPCs, and its timely downregulation is required for remyelination (**6** [195, 200]. The small heat shock protein HSPB5 (CRYAB) is upregulated in WM but not in GM MS lesions (**7** [275, 276]). ASTRs are indicated with a yellow border and oligodendroglial lineage cells with a blue border

Regional differences in remyelination efficiency in experimental rodent models may be explained by the intrinsic differences between OPCs, which may be acquired during development. For instance, during the third OPC wave in the developing brain, the corpus callosum is mainly populated by cortex-derived dorsal oligodendroglial lineage cells, and only 20% of the oligodendroglial lineage cells in the adult corpus callosum are from the ventral forebrain [122]. Upon toxin-induced demyelination in the corpus callosum, these dorsal-derived OPCs in the corpus callosum have a higher remyelination capacity than the ventral-derived OPCs and display an enhanced capacity to migrate and differentiate in vitro [206]. Also, upon cuprizone-induced demyelination, the expression of G-protein coupled receptor 17 (GPR17) is induced by OPCs in the corpus callosum, but not by OPCs in the cortex [207] (Fig. 3). In the corpus callosum, GPR17 is expressed by maturing oligodendroglial lineage cells, where it is involved in the initiation of differentiation [202]. Timely downregulation of GPR17 is required for terminal OLG differentiation and myelination. Hence, GPR17 may play a central role in orchestrating repair processes in WM, but not in GM, including remyelination [208]. Importantly, rodent adult OPCs respond to demyelinating injury by reverting to a less complex morphology [209, 210] and a more immature state at the transcriptional level [54] before differentiating and, ultimately, remyelinating denuded axons. In addition, activated adult OPCs display increased migratory properties and accelerated differentiation compared to resting adult OPCs [54]. Moreover, activated adult OPCs directly regulate their recruitment to demyelinated areas by increasing their expression of IL1β and CCL2 [54]. Notably, regional differences were not taken into account and IL1β and CCL2 expression is only verified in oligodendroglial lineage cells in the corpus callosum [54]. In reverting to a more immature state, gmOPCs may have an advantage, as gmOPCs exert a less complex morphology than wmOPCs in vitro [95, 101], and are already less mature at the gene expression level [101]. Moreover, in vitro gmOPCs are less sensitive than wmOPCs to the detrimental effects of the inflammatory mediator IFNγ on proliferation, differentiation and morphology, and migrate more in response to ASTR-secreted factors [101]. Also, growth factors that affect OPC behavior, including CNTF, BDNF, FGF2, and HGF, are differentially expressed upon GM and WM demyelination. Taking temporal expression into account, during cuprizone-induced demyelination, the expression of these growth factors is upregulated during remyelination in the corpus callosum, while they are not required for remyelination in the cortex (CNTF, BDNF) or are preferentially expressed during demyelination in the cortex (FGF2, HGF) [211]. Notably, CNTF and BDNF accelerate OPC maturation [212, 213] and FGF2 and HGF both enhance OPC proliferation and migration and prevent their differentiation [46, 214, 215]. Thus, remyelination efficiency depends on intrinsic differences between gmOPCs and wmOPCs as well as on the availability of signaling factors, such as growth factors, to respond to. While differences in gmOPC and wmOPCs responses towards demyelination-relevant injury signals are evident, differences between responses of the distinct mature myelinating OLG populations towards CNS injury have not been reported yet. As ASTRs are the cellular source of CNTF [216], BDNF [217], HGF [218], and FGF2 [219], ASTR diversity may contribute to the differences in remyelination efficiency in GM and WM.

### Astrocyte diversity and remyelination

In addition to regional diversity of OPCs, ASTR responses towards injury may also vary between regions. Upon OLG and myelin loss, ASTRs become reactive, which in the cuprizone model involves ASTR proliferation, upregulation of reactive ASTR markers such as GFAP and vimentin, and the elaboration of a dense network of processes [19, 20, 204, 211, 220, 221]. In experimental demyelination models, ASTR reactivity is more prominent in the corpus callosum than in the cortex [19, 20, 204, 211, 220, 221], although ASTR reactivity has been suggested to start earlier in the cortex [222]. ASTR reactivity is regulated by pro-inflammatory cytokines, Toll-like receptor (TLR)-mediated signaling events, and myelin debris [220, 223–226]. As the BBB remains virtually intact in the cuprizone model [227], most inflammatory mediators that induce ASTR reactivity are provided by microglia. In the cuprizone model, microgliosis precedes loss of OLGs and is in the corpus callosum already apparent when myelin still appears normal [222]. In contrast, in the cortex, microglia activation is less prominent and delayed [222]. Hence, early microglia activation precedes ASTR reactivity in the corpus callosum, while ASTR reactivity in the cortex is already evident when microglia activation peaks. This indicates that ASTR reactivity upon GM and WM demyelination is heterogeneous as a consequence of differential inducing signal factors. Of note, both in the corpus callosum and cortex, transcripts of the chemokine CCL2 are transiently enhanced early upon cuprizone administration, while mRNA levels of CCL3 continuously increase [222]. However, when CCL2 and CCL3 are both absent, both ASTR reactivity and demyelination in the cortex but not in the corpus callosum are reduced [228]. This is in line with the assumption that ASTR reactivity differs in GM and WM and therefore distinctly modulate de- and remyelination. Consistent with this, ASTR reactivity is heterogeneous, and depends on the type of injury and the inducing mediator(s) [226, 229]. Reactive ASTRs have been classified as anti-inflammatory A2-ASTRs, induced by myelin debris [224] and/or TLR3 agonists [225, 230] and characterized by S100A2 expression [224], and pro-inflammatory A1-ASTRs induced by microglia-derived IL-1α, TNF and C1q and characterized by C3 expression [88, 224]. Mild activation of ASTRs may induce pro-reparative A2-ASTRs, while the more reactive A1-ASTRs inhibit OPC proliferation, migration and differentiation and secrete toxic factors for OLGs [224, 231–233]. Notably, transgenic overexpression of GFAP alters the chemokine secretory profile of ASTRs and protects against cuprizone-induced demyelination in the corpus callosum [234], indicating that ASTR reactivity that is correlated with an upregulation of GFAP may serve a protective function. The authors did not report on differences in GM.

Another feature of reactive ASTRs is increased deposition of ECM proteins. Upon toxin-induced demyelination, ASTRs transiently deposit several ECM proteins, including CSPGs and fibronectin, which add to resolve injury and promote recovery [221, 235–239]. The composition of the ECM affects OPC behavior; fibronectin increases OPC proliferation and migration and inhibits OPC differentiation [236, 239–248], while CSPGs inhibit OPC proliferation, migration and differentiation [239, 249–253]. Differentiation of neural stem cells into OPCs and finally into mature myelinating OLGs is, in addition to ECM composition, also dependent on the stiffness of the ECM [254]. A rigid matrix promotes OPC proliferation and early differentiation, while a soft matrix favors OLG maturation and myelination [254]. Regional differences in stiffness have been observed; WM is more stiff compared to GM which is, among others, due to a higher abundance of myelin [255]. Notably, in the cuprizone model, a decreased stiffness in the corpus callosum is observed upon acute demyelination, while in chronically cuprizone-induced demyelinated lesions that fail to remyelinate an increase in ECM deposition and tissue stiffness is measured [235]. Therefore, enhanced deposition of ECM proteins in the corpus callosum may contribute to recruitment and early differentiation of OPCs, but removal of these ECM proteins is required for OLG maturation and myelination. ECM proteins are degraded, among others, by metalloproteinases (MMPs), which are mainly expressed by microglia and ASTRs [256]. In the cuprizone model, ASTRs in the corpus callosum express both MMP3 and MMP12 during remyelination, while hardly or no expression of these MMPs was detected in ASTRs in the cortex [256]. This indicates that ECM remodeling by these MMPs is more relevant in WM than in GM during remyelination. Hence, it is tempting to suggest that a regional difference in inducing stimuli and ECM remodeling by ASTRs during reactive gliosis [19, 221] may add to local differences in remyelination efficiency in the cortex and corpus callosum.

A potential role of pre-existing heterogeneity of gmASTRs and wmASTRs in myelination efficiency has recently gained more evidence. Both in vivo and in vitro studies have shown that ASTRs support (re)myelination by supplying lipids, including unsaturated fatty acids and cholesterol, to OLGs [61, 62]. Strikingly, when blocking lipid biosynthesis in ASTRs during development, hypomyelination is more evident in WM than in GM [61], indicating that developmental myelination in WM depends more on ASTR-derived lipids. In addition, primary gmASTRs export more cholesterol and are more supportive for in vitro myelination than wmASTRs [257]. Hence, while myelination in WM relies more on lipids supplied by ASTRs, gmASTRs actually appear better equipped for the supply of cholesterol. Surprisingly, inhibition of committed cholesterol biosynthesis in wmASTRs but not gmASTRs, increases in vitro myelination [257]. As cholesterol biosynthesis is intertwined with unsaturated fatty acid and non-sterol isoprenoid biosynthesis [258–260], their upregulated synthesis upon blocking committed cholesterol synthesis may have obscured the effect of decreased cholesterol levels. In fact, an increase in non-sterol isoprenoid synthesis increases isoprenylation, which reduces the release of pro-inflammatory cytokine IL1β from cells, including ASTRs [257, 261] and likely also the release of other cytokines [262]. Therefore, modulating lipid biosynthesis in wmASTRs but not gmASTRs, alters the inflammatory microenvironment in WM, which affects wmOPC differentiation.

Taken together, in experimental models, the regional difference in remyelination efficiency may be explained by pre-existing OPC and ASTR heterogeneity as well as plasticity, which thus depends on the context of injury and local inducing stimuli. Whether macroglial diversity and their interactions may also play a role in remyelination efficiency in GM and WM MS lesions will be described next (summarized in Fig. 3).

## Macroglial diversity and its relevance for multiple sclerosis

### Remyelination in multiple sclerosis lesions

MS is a chronic inflammatory and progressive disease of the CNS characterized by the formation of demyelinated lesions that, upon failure of remyelination, ultimately lead to neurodegeneration and an increasing state of neurological disability [263]. Substantial remyelination is reported to occur at any given age, even well into the 8th decade of life [7, 264–266]. However, remyelination efficiency is variable; lesions are most efficiently repaired in the early stages of MS, while remyelination is often limited upon aging and disease progression [201, 264, 265, 267]. More remyelinated lesions are detected in progressive MS than in RRMS, and the proportion of remyelination is lower in patients with cortical GM lesions [267]. Also, MS patients with a shorter disease duration have a smaller proportion of remyelinated lesions [267]. Possible explanations for the decrease in remyelination efficiency include failure of OPC recruitment to the lesion, failure of OPC differentiation into myelinating OLGs, and/or failure of OLGs to effectively remyelinate axons [9, 250, 268–271]. In 70% of WM MS lesions, OPCs are present but fail to remyelinate denuded axons [250, 268, 269]. This indicates that remyelination is often not limited by an insufficient amount of OPCs, but rather by a failure of OPC differentiation [268]. Recent snRNAseq studies confirmed that OPCs in MS lesions are indeed relatively quiescent on a transcriptional level [120, 121]. Experimental toxin-induced demyelination models revealed that the speed of remyelination, as other regenerative processes, decreases with age [9, 272]. OPC characteristics affected by aging may contribute to impaired OPC differentiation. For example, CREB signaling in OPCs is impaired upon aging in a mouse model of prolonged WM cerebral hypoperfusion [273]. A recent study on OPCs obtained from whole rat brain revealed that aged OPCs acquire classical hallmarks of cell aging, including increased DNA damage, decreased metabolic function, and become irresponsive to pharmacological-applied differentiation signals, such as miconazole and benzatropine [56]. The observation that myelination in the adult CNS is accompanied by more and shorter myelin segments, and that the produced myelin is thinner, is also observed in remyelinated MS lesions [274]. This may imply that this is a feature of adult myelination, rather than an impaired myelin phenotype in remyelination [48]. Remarkably, carbon dating studies on WM brain tissue revealed that newly-formed OLGs, i.e., generated from adult OPCs, were only detected in a small subgroup of patients that had an aggressive form of MS [155]. Intriguingly, in WM-derived shadow plaques, i.e., remyelinated areas [275], newly-formed OLGs were absent, indicating that remyelination is not performed by adult OPCs, but by mature pre-existing OLGs generated during development [155]. This is in line with an electron microscopy study in disease models of cats and non-human primates that uncovered that mature OLGs are connected to myelin sheaths of different thickness, indicating that the myelin sheaths are generated during both development and remyelination [276]. Whether the contribution of ‘old’ pre-existing mature OLGs to remyelination is specific to WM, or whether this is an adaptation, i.e., a ‘gain-of-function’ of mature OLGs as a consequence of the quiescent OPCs in pathological lesions, remains to be determined.

### Differences in remyelination of grey and white matter multiple sclerosis lesions

Historically, MS was considered mainly a disease of the WM, but now it has been well recognized that also GM pathology is prominent. The distinct WM lesions are classified by demyelination and inflammatory activity [264, 267], while GM lesions are categorized on basis of their location. WM MS lesions are characterized by a variable infiltration of lymphocytes and macrophages, glial scar formation, and microglia activity [267, 277–280]. By contrast, GM MS lesions are characterized by the loss of OLGs in the presence of a seemingly intact BBB, axonal loss, mild astrogliosis, and reduced lymphocyte and macrophage infiltration [281–283]. Hence, as in toxin-induced demyelination models, GM and WM demyelination and remyelination may differ due to differences in the abundance and timing of inducing signaling factors. To study regional differences in remyelination, leukocortical lesions, i.e., lesions that span both GM and WM areas, are therefore of special interest, as these lesions are assumed to have a similar pathological background and age when comparing GM to WM. Similar to what is observed in the cuprizone model [19, 20], the GM cortical portion of leukocortical MS lesions has a higher remyelination capacity than the WM non-cortical part [7] (Fig. 3). In addition, the OLG density is 6.8 fold higher in the GM part than the WM part [7]. Also, the number of NG2-positive cells, which are mainly OPCs, is reduced in WM MS lesions compared to normal appearing WM (NAWM) (40–80/mm^2^ *versus* 140–150/mm^2^) [110]. The lower abundance of OPCs in WM MS lesions may be below the threshold for successful remyelination. In contrast, the number of OPCs is comparable between normal appearing GM (NAGM) and demyelinated GM MS lesions (98/mm^2^ *versus* 110/mm^2^), and even higher than in control GM (63/mm^2^) [7]. Furthermore, astrogliosis and the expression of OPC differentiation-inhibiting ECM components are higher in the WM part than in the GM part of leukocortical MS lesions. In addition, microglia are also more reactive in the WM part, possibly contributing to the differential effect on remyelination [7]. These findings are not restricted to leukocortical lesions, i.e., also in non-leukocortical GM MS lesions remyelination is more pronounced than in WM MS lesions [8, 280]. Transcriptome analysis on post-mortem tissue of different brain regions of MS patients and healthy subjects identified more disease-related changes in the corpus callosum than in the cortex most prominently in myelinating OLGs, but also in ASTRs [284], hinting at diversity of OLG responses. Taken together, OLGs, but not OPCs, display the most intrinsic regional heterogeneity, while differences in ASTR reactivity mostly depend on the presence of injury signals. These injury signals likely differ in GM and WM, including the amount of myelin debris, and the presence of reactive microglia. Which molecular differences underlie OLG diversity in MS lesion pathology is discussed next.

### Oligodendroglial lineage cell diversity in white matter multiple sclerosis lesion pathology

Two independent snRNAseq studies on post-mortem WM brain tissue found significant differences in the transcriptome of mature OLGs in WM MS lesions compared to WM tissue of healthy subjects, while OPCs were transcriptionally quiescent [120, 121]. More specifically, of the six identified mature hMOL populations in control human brain tissue, the fully mature hMOL1 that does not express high levels of myelination related genes and the more immature hMOL6 population were less abundant in WM MS lesions, while imOLG, hMOL2, hMOL3 and hMOL5 were enriched in WM MS lesions (Fig. 2b). Thus, in WM MS lesions, hMOLs populations were skewed to the transcriptionally different fully mature hMOL5 population and/or other populations were depleted. In favor of the latter is that in MS lesions adult OPCs lack the capability [120] and/or receive inhibitory signals [201, 239] to form new hMOLs, which may represent the reduced abundance of the pre-myelinating hMOL6 [121]. In addition, increased transcript levels of myelin genes were observed in mature wmOLGs in MS [121], hinting to the involvement of mature OLGs in remyelination. Spatial analysis of MS lesions uncovered that genes related to OPC differentiation were reduced at the border of WM lesions and that genes belonging to stress pathways and related to iron accumulation were increased at these borders [120]. Although not studied [121, 155], nor extensively discussed yet [120], given that OPCs are more abundant in GM MS lesions [7, 110] and the selective appearance of stressed OLGs at WM lesion borders [120], it is tempting to suggest that remyelination in GM may proceed via newly-formed OLGs and remyelination in WM MS lesions via pre-existing OLGs.

Both snRNAseq studies on MS brain tissue revealed an immunocompetent phenotype in oligodendroglial lineage cells at all maturation stages, and include increased transcript and protein levels of major histocompatibility complex class I (MHC-I) [120] and MHC-II [183]. An immunocompetent phenotype of both OPCs and OLGs is also present in experimental autoimmune encephalomyelitis (EAE) [183], an animal model that resembles autoimmune inflammatory aspects of MS. scRNAseq of spinal cord (spc)-derived oligodendroglial lineage cells at the peak of EAE identified eight mature spc-OLG populations, of which five are EAE-associated, including a spc-mMOL population that mainly comprises newly formed OLGs [183] (Fig. 2a). Further analysis identified an EAE-specific gene module containing genes that were more abundantly expressed by the newly formed OLG population and another module that comprised genes associated with the IFN response pathway and MHC-I and MHC-II genes (Fig. 2a) [183]. Strikingly, in contrast to control spinal cord tissue [53, 119, 183] where only one spc-mOPC population was present, three additional spc-mOPC populations were observed in EAE tissue. Of the three EAE-specific spc-mOPC populations, one was a cycling spc-mOPC population, whereas transcripts of myelination related genes are increased in the other two spc-mOPC populations. This indicates that these EAE-associated spc-mOPCs were transferred from a quiescent state to an actively differentiating state [183] (Fig. 2a). Notably, transcriptionally different OPC populations are not detected in WM MS lesions [120, 121], indicating that in contrast to EAE, OPCs are likely not activated and triggered to differentiate in MS. Alternatively, given that most post-mortem MS lesions are likely relatively old, and may have had their initial demyelinating event months to years in the past, it cannot be excluded that in newly-emerged MS lesions OPCs are more active. Surprisingly, EAE-associated spc-mOPCs also express MHC-II genes of which induction is mediated by IFNγ in vitro [183]. IFNγ also induces MHC-I expression in gmOPCs, and these MHC-I expressing gmOPCs present antigens to cytotoxic T cells [285]. Given that wmOPCs are more susceptible to IFNγ than gmOPCs in vitro [101], it would be interesting to investigate whether wmOPCs also display MHC-reactivity upon exposure to IFNγ. Of relevance, EAE-associated spc-mOPCs exhibit phagocytic activity, take up myelin debris and likely present myelin-specific antigens [183]. Of note, bulk-RNAseq of OPCs revealed an upregulation of genes associated with the innate immune system, such as IL1β and CCL2 upon cuprizone-induced demyelination [54], but not an upregulation of MHC-I or MHC-II genes in adult OPCs. Hence, it would be interesting to investigate with scRNAseq whether upon cuprizone-induced demyelination distinct OPC clusters can be identified.

In conclusion, the upregulation of immunomodulatory genes in oligodendroglial lineage cells suggests that these cells may have a more direct role in MS disease origin and progression, and contribute to OLG heterogeneity. Alternatively, the upregulation of immunomodulatory genes in oligodendroglial lineage cells may represent a natural, transient response towards inflammation-mediated demyelination, but persists in MS. In addition, in EAE but not MS, OPCs are transcriptionally active, suggesting that transcriptional activation of OPCs in MS lesions is impaired.

### Astroglial scar formation white matter multiple sclerosis lesions

ASTRs change their phenotype in demyelinated MS lesions and astrogliosis varies between GM and WM MS lesions. Phenotype clustering of ASTRs and myeloid cells with the use of mass cytometry and thirteen glia-related markers revealed the presence of five different types of ASTRs in MS lesions [286]. Two of these were present in the center of GM and WM lesions, one on the inner GM and WM rim and one on the WM outer rim, and the final subtype of ASTRs was present in NAWM. As the ASTR phenotypes localize to different zones of MS lesions, it is suggested that these phenotypes are functionally diverse populations [286]. In line with the snRNAseq studies that identified only two to three ASTR subpopulations [120, 121, 183, 287], the five different ASTR populations may be a representation of functional plasticity of the same ASTR subtype in MS, rather than a representation of intrinsic ASTR heterogeneity. The two ASTR populations identified by snRNAseq both expressed *RFX4* and represent protoplasmic ASTRs, in MS lesions characterized by a downregulation of *SLC1A2*, and fibrous/reactive ASTRs that express more *GFAP*, *CRYAB* and *MT3* in MS tissue [120]. This indicates that astrogliosis is more apparent in WM MS lesions than in GM MS lesions. Indeed, the small heat shock protein CRYAB, also named HSPB5, supports the reactive ASTR response that contributes to demyelination in the cerebellum of the cuprizone model [288] and is upregulated in active and chronic WM MS lesions, but not in GM MS lesions in both brain and spinal cord [289, 290] (Fig. 3). A recent study that combines scRNAseq, Ribotag RNA profiling, ATAGseq and ChIPseq identified an EAE disease-associated *Gfap*^+^ ASTR subpopulation that expressed higher levels of MAFG and lower levels of antioxidant NRF2 target genes and that promoted inflammation during EAE [291]. A similar ASTR signature is found in WM MS lesions [291], indicating a robust machinery and possibly allowing for therapeutic intervention overcoming ASTR-mediated inflammation and oxidative stress in MS.

Together with a dense network of ECM proteins, hypertrophic ASTRs form a so-called astroglial scar around inflammatory WM lesions but not GM lesions. This astroglial scar consists of new, proliferative ASTRs, which no longer occupy discrete microdomains and instead have overlapping processes that form a barrier against inflammation [231]. The astroglial scar in MS lesions is usually considered detrimental for remyelination [292, 293]. In contrast, even though reactive ASTRs do emerge in the corpus callosum of the cuprizone model even beyond the demyelination period, the dense network of ASTR processes do not progress to form a barrier along the lesion upon prolonged cuprizone feeding [221]. Possibly, as a consequence of a distinct inflammatory profile, and locally expressed inducing stimuli in GM and WM MS lesions, ASTR reactivity is increased in WM, a difference that is particularly evident in leukocortical lesions [7]. It would be interesting to investigate whether the reactive astrogliosis and the formation of a glial scar in WM MS lesions may account for decreased presence and/or differentiation to the fully mature hMOL1 as identified by the snRNAseq study [121].

The astroglial scar in WM lesions mainly consists of interwoven astrocytic processes [294]. The processes of the ASTRs in the glial scar are highly filamentous, expressing high levels of GFAP, vimentin, and nestin [295]. A function of a glial scar is to prevent spreading of inflammation to adjacent tissue, thus limiting further tissue damage (reviewed in [294]). At the edges of active and expanding WM MS lesions, CSPGs are produced by ASTRs [296] under control of regulator TRPM7 [297]. The reduced remyelination capacity in WM has been correlated with the accumulation of the CSPG versican, which is expressed by wmASTRs but not by gmASTRs in leukocortical lesions [7]. A high number of cells positive for NG2 (CSPG4) is found at the edge of the glial scar in (chronic) active MS lesions [110]. Although often used as a marker for OPCs, NG2-expressing cells can also become ASTRs in vivo [105], and in rodents also microglia initiate NG2 expression upon aging [298]. Other ECM proteins that impair OLG production and remyelination include hyaluronan and fibronectin [7, 236, 239, 299–301]. Hyaluronan and its receptor CD44 are significantly increased in the WM, but not in the GM part of leukocortical lesions [7]. Fibronectin is transiently expressed in toxin-induced demyelination models and aids OPC recruitment, while newly-formed OLGs only maturate upon fibronectin clearance [221, 236, 242, 302–304]. However, while undetected in toxin-induced demyelination, ASTRs form remyelination-impairing fibronectin aggregates in WM MS lesions [236]. Aggregate formation is likely induced by insufficient fibronectin degradation [305] combined with chronic inflammation [236, 305, 306]. Fibronectin aggregates persist in WM MS lesions, impairing OPC differentiation and thereby contributing to remyelination failure [236, 304]. Although GM MS lesions have not been studied in the context of fibronectin aggregates yet, fibronectin is not present in GM marmoset EAE lesions [237]. Also, in vitro more fibronectin aggregates are formed by wmASTRs than by gmASTRs and may reflect intrinsic differences in alternative splicing of fibronectin between gmASTRs and wmASTRs [306]. Alterations in ECM composition is reflected in tissue stiffness, which may contribute to the regional difference in remyelination efficiency in MS lesions. In favor of this hypothesis, tissue stiffness is transiently decreased in acute demyelinated WM MS lesions and experimental toxin-induced demyelination models [235], thus forming an environment which supports active myelination [254]. In contrast, chronic demyelinated WM MS lesions are stiffer than control tissue [235] (Fig. 3), thus providing a myelination-inhibiting environment [254]. Regional differences in stiffness dynamics have also been reported in the chronic EAE model for inflammation-mediated demyelination [307]. Contrasting to chronic cuprizone and MS WM lesions, in EAE WM, an initial increase in stiffness is observed at EAE onset and peak phase of demyelination, which is followed by a decrease in stiffness in the chronic phase, while GM stiffness remains unaffected [307]. The latter may be a reflection of the absence of GM pathology in murine EAE.

### Astrocyte signaling in grey and white matter multiple sclerosis lesions

OPCs are absent in 30% of WM MS lesions [250, 269, 270], indicating a failure of OPC recruitment. Semaphorins are extracellular signaling guidance proteins that play an important role in OPC recruitment upon toxin-induced demyelination. Semaphorin 3F (Sema3F) acts as a chemo-attractant and Sema3A as a chemo-repellent for OPCs [308]. In active WM MS lesions, Sema3A and Sema3F expression is increased in both reactive ASTRs and microglia [309, 310]. Whereas Sema3F expression is abundant in active WM lesions with a high load of microglia/macrophage infiltration, Sema3A expression is predominant in lesions with less infiltrated microglia/macrophages [310]. Both semaphorins are also detected in GM MS lesions, but in GM their expression is strictly neuronal [310], indicating that semaphorins are differentially expressed by gmASTRs and wmASTRs. Upon toxin-induced demyelination in the spinal cord, lentivirally mediated overexpression of Sema3A in ASTRs inhibits OPC recruitment to demyelinated areas and thereby prevents remyelination [311]. When Sema3A is injected into focal toxin-induced demyelinated lesions, OPC differentiation is halted at the pre-myelinating stage [309]. These pre-myelinating OLGs contact axons but fail to produce myelin sheaths. Thus, Sema3A in MS lesions may in addition to diminishing OPC recruitment, also halt differentiation of OPCs in a pre-myelinating stage. Of note, in the WM part of leukocortical lesions, pre-myelinating OLGs with multiple processes that are associated with demyelinated axons are present but fail to myelinate the denuded axons [7]. As gmASTRs do not express Sema3A, and as the expression of neuronal Sema3A does not a play major role in OPC recruitment [309], the differential expression of semaphorins in gmASTRs and wmASTRs may contribute to increased remyelination efficiency in GM lesions. This is consistent with the abundant presence of OPCs in GM MS lesions [7, 8].

Direct cell–cell contact via gap junctions between ASTRs and OLGs is altered in MS lesions. In both GM and WM MS lesions, Cx43 coupling between ASTRs is increased, which is also observed in NAGM, but not in NAWM [312, 313]. Also, gap junction formation between GM-specific Cx30s is upregulated in ASTRs in GM MS lesions [313]. In contrast, in EAE, a loss of Cx43 expression in ASTRs is observed in the spinal cord [314]. Deletion of Cx43 or blocking Cx43 hemichannel activity, i.e., independent of gap junctional communication, positively affects remyelination by reducing local activation of ASTRs and favoring the clearance of myelin debris and the generation of pro-regenerative microglia, thereby enhancing OPC maturation [315]. Gap junction formation with OLG Cx32 and OLG Cx47 is reduced in GM and WM MS lesions, with extension of the loss of Cx32 into NAGM and NAWM [313]. Cx47 expression is however increased in a number of OPCs in NAWM, although these OPCs have limited coupling to ASTRs [312, 313] (Fig. 3). Possibly this limited ASTR/OPC coupling is related to the failure of OPCs to differentiate into pre-myelinating OLGs [198, 313]. Similar as in healthy GM where gap junctions between ASTRs and OLGs seem less important for OLGs and myelin maintenance than in WM [198], it is tempting to hypothesize that remyelination in GM lesions may also be less affected by the loss of connexin expression by OLGs. Upon cuprizone-induced demyelination in the corpus callosum, Cx47 is initially upregulated in OLG processes, and in parallel a transient and de novo expression of Cx47 is observed in ASTRs [316]. Upon remyelination, Cx47 expression is switched back from ASTRs to the newly-formed OLGs [316]. Notably, ASTR signaling via Cx47 coupling to OPCs increases the expression of sphingosine-1-phosphate receptor 3 (S1PR3), which is activated by the lipid signaling molecule sphingosine-1-phosphate (S1P), and thereby promotes OPC proliferation [317]. Thus, the increased expression of Cx47 in OPCs in NAWM may aid proliferation of OPCs, which is in line with the observed increase in OPC numbers in NAWM [312]. On the other hand, ASTRs express S1PR1, also a receptor for S1P. In acute GM lesions, S1PR1 expression is decreased on ASTRs, while it remains present on ASTRs in WM lesions. Of importance, in GM MS lesions, S1PR1 reappears on ASTRs when inflammation diminishes [318]. Moreover, the activation of S1PR1 on ASTRs promotes inflammation [319], indicating that expression of S1PR1 plays a role in inflammation of WM but not GM MS lesions. Notably, treatment with fingolimod, an inhibitor of S1PR, ameliorates EAE among others by inhibition of inflammatory activation of ASTRs and recovery of BBB function [319, 320]. Hence, ASTR/OPC and ASTR/OLG interactions are distinct in GM and WM and may contribute to regional differences in remyelination efficiency in GM and WM MS lesions and are of relevance for assessing therapeutic effects.

## Concluding remarks and perspectives

After initially being described well over a century ago, macroglial diversity has gained interest and momentum in the last few years, which is not only relevant for understanding human pathology, including MS, where regional differences in remyelination efficiency are evident, but also for the design of therapeutic strategies. While OPCs appear transcriptionally relatively homogenous [53, 119–121], clear functional differences between gmOPCs and wmOPCs exist, both in vitro and in vivo [30, 51, 52, 94, 96–101, 104–106]. This may be attributed to regional differential aging of either OPC population and as a result renders gmOPCs likely better equipped for remyelination. Transcriptional profiling further reveals that oligodendroglial lineage cells become more heterogeneous upon maturation and may specialize in different location-dependent functions [53, 119–121]. However, the exact function of these distinct populations is not completely clear. In this review, we have mostly focused on the effects on myelination and remyelination, and whether the more robust remyelination in GM than in WM [7, 8, 19, 20, 211] is an indication of the significance of differences in macroglia from different brain regions for remyelination. However, other functions of oligodendroglial lineage cells in synaptic pruning and metabolic support of axons may also be differentially regulated between different brain regions and remains to be determined. Also, the significance and function of the gain of an immuno-oligodendroglial lineage phenotype that is observed in MS and in EAE lesions [183, 285] in the context of remyelination (failure) between regions requires further exploration. Furthermore, in contrast to experimental models where remyelination is performed by newly-formed OLGs, remyelination in WM MS lesions may be performed by pre-existing, mature OLGs [155]. Therefore, obtaining more insight into the function of the two transcriptionally distinct mature OLG populations that are reduced in WM MS brain tissue [121] is of interest, as well as whether transcriptionally different mature OLGs exists in GM and GM MS lesions, which may contribute to regional differences in the extent of remyelination and MS pathology.

In contrast to mature OLGs, ASTRs appear less heterogeneous on a transcriptional level, i.e., in RNAseq studies, only two to three groups of transcriptionally different ASTRs were identified [121, 138, 183, 184]. However, ASTRs display high plasticity and adapt to the specific demands of the local cells and injury functional needs [65, 66], which may result in subsequent ASTR regional diversity [321, 322]. For example, demyelination in GM and WM induce different types of injury responses, with distinct local induced factors [323, 324], and as a consequence differences in ASTR reactivity. Hence, whether and how regional diversity and interplay between macroglia from the GM and WM contribute to observed differences in remyelination efficiency and MS pathology is currently difficult to dissect. Therefore, more research both in vitro and in vivo is required to determine whether gmASTR and wmASTRs respond differently to similar microenvironmental signals. For example, to exclude that the remyelination process is mainly dictated by the resident microenvironmental signals, homo- and heterotopic transplantation of either ASTR in demyelinated GM or WM may be considered. Such transplantation studies have been performed for gmOPCs and wmOPCs in healthy adult brains, clearly hinting at intrinsic regional differences of OPCs in their maturation capacity [95]. Transplantation of OPCs derived from different brain regions into demyelinated areas have not been reported yet, but given that gmOPCs are less mature and less susceptible to inflammatory cytokines, they may be better equipped for remyelination than wmOPCs [54, 101]. Co-cultures of primary OPCs and ASTRs will also be valuable in understanding the role of macroglial diversity on the interplay between OPCs and ASTRs from different regions and its effect on myelination. The role of microglia and infiltrating immune cells in regional differences in remyelination efficiency have not been thoroughly discussed in this review, but are likely to be important players in inducing different type of injury response in GM or WM [19, 324–327]. Also, the lower levels of myelin (debris) in GM [328] and the putative differences in myelin composition in GM compared to WM (Table 1) may contribute to regional differences in remyelination efficiency [158, 159].

Finally, many in vitro studies use GM-derived macroglia, while experimental models often focus on WM regions. The ignorance of macroglial diversity may lead to conflicting results obtained by in vitro and in vivo studies. Therefore, when studying the effect of potential remyelination promoting therapeutics, research should take into account region-specific outcomes. For example, modulating committed cholesterol synthesis is supportive for in vitro myelination when blocked in wmASTRs while having no effect when blocked in gmASTRs [257]. Although not studied yet, this may imply that enhancing committed cholesterol synthesis in ASTRs may actually be detrimental for remyelination in WM but not in GM. Hence, more refined approaches taking macroglial diversity into account will be beneficial for myelin research, and for the identification of therapeutic targets that may promote remyelination, particularly in WM MS lesions where remyelination is less effective.
